# Sensitivity-Enhanced Temperature Sensor Based on PDMS-Coated Mach–Zehnder Interferometer

**DOI:** 10.3390/s25041191

**Published:** 2025-02-15

**Authors:** Wenlei Yang, Le Li, Shuo Zhang, Ke Tian

**Affiliations:** 1 College of Information and Intelligence Engineering, Zhejiang Wanli University, Ningbo 315100, China; yangwenlei@hrbeu.edu.cn; 2 Huazhong Institute of Electron-Optics, Wuhan National Laboratory for Optoelectronics, Wuhan 430223, China; 3 College of Physics and Optoelectronic Engineering, Harbin Engineering University, Harbin 150001, China; ketian@hrbeu.edu.cn

**Keywords:** Mach–Zehnder interferometer, fiber-sensing technology, polydimethylsiloxane, temperature measurement

## Abstract

A sensitivity-enhanced temperature sensor based on a Mach–Zehnder interferometer (MZI) coated by polydimethylsiloxane (PDMS) film is proposed and investigated. The MZI with a compact size of 2.28 mm is fabricated by embedding a tapered single-mode fiber (SMF) between two multimode fibers (MMFs). Since PDMS has a higher thermo-optical coefficient than silica, the proposed sensor has better temperature sensing performance than the case without PDMS coating, which is demonstrated by simulation and experiment. The experimental results show that the sensitivity of the proposed sensor is as high as −1.06 nm/°C in the range from −5 °C to 45 °C.

## 1. Introduction

Temperature assessment plays an important role in various fields, such as crop production, environmental monitoring, food storage, and medical diagnosis [1,2,3,4,5,6]. In the past two decades, a wide variety of optical fiber temperature sensors have been extensively studied due to the advantages of compactness, being lightweight, high temperature resistance, and anti-electromagnetic interference [7,8,9,10]. For example, Wu et al. prepared a Fabry-Perot interferometer temperature sensor with a sensitivity of 13 pm/°C by splicing a piece of photonic crystal fiber to a standard single-mode fiber [11]. Zhang et al. proposed a fast response temperature sensor based on fiber Bragg gratings. Although the response time could be as low as 48.6 ms, the temperature sensitivity was only 27.6 pm/°C [12]. In 2021, Liu et al. presented a high temperature fiber-optic sensor (up to 1000 °C) with a sensitivity of 48.2 pm/°C by fusing a section of few-mode fiber between two single-mode fibers [13]. In 2023, Wang et al. reported an anti-interference temperature sensor by fusing a home-made Kagome hollow-core photonic crystal fiber (PCF) with two no-core fibers and two single-mode fibers; the sensor had a maximum temperature sensitivity of 38.39 pm/°C [14]. However, the temperature sensitivity of the sensors mentioned above is difficult to enhance due to the low thermal optical coefficient (TOC) of quartz fiber (the TOC of silica is 5.5 × 10^−7^/°C) [15]. Therefore, the traditional silica fiber-based temperature sensor configuration needs to be improved.

Researchers are constantly exploring and innovating methods to improve the sensitivity of optical fiber temperature sensors. In general, these efforts can be categorized into three main approaches for improving temperature sensitivity. The first method is to introduce special optical fibers into the fiber optic sensor, such as hollow core fiber [16], polarization maintaining fiber [17], photonic crystal fiber [18], and so on. By combining the microstructure or sensing mechanism of the special fiber, the sensitivity of the temperature sensor can be improved. The second method is to sensitize in combination with the optical vernier effect by measuring the response of the vernier spectral envelope [19,20]. The third method is to introduce thermosensitive materials into the fiber optic sensor, combining their high thermal optical coefficient or high thermal expansion coefficient for enhanced sensitivity. Recently, various sensors based on high TOC materials [21,22,23,24], such as alcohol liquid, refractive index matching liquid, isopropanol liquid, and glycerol–water solution, have been adopted for temperature measurement. Although the sensitivity of these sensors has been effectively improved, their preparation process is complicated and the encapsulated high TOC liquid is prone to volatilization or leakage, resulting in a relatively limited lifespan for these sensors [25,26].

In this article, we demonstrate a highly sensitive temperature sensor based on a Mach–Zehnder interferometer coated with PDMS. Owing to the thermo-optical and thermal expansion effects of PDMS, the change in temperature can be judged by analyzing the resonance wavelength shift in the transmission spectrum. The experimental results show that the proposed sensor has a temperature sensitivity of up to −1.06 nm/°C in the range from −5 °C to 45 °C. Furthermore, as an interference-based MZI sensor, its total length is only 2.28 mm. The PDMS-coated MTSM, characterized by high sensitivity and compact size, emerges as a crucial choice for temperature sensing applications.

## 2. Fabrication and Principle

**Figure 1:**
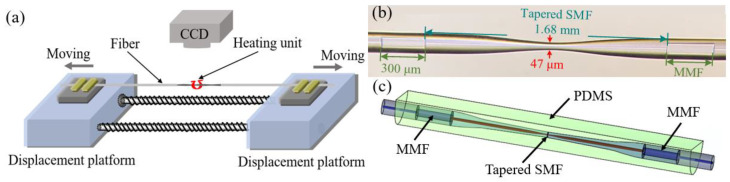
(**a**) Schematic illustration of the fusion tapering device; (**b**) optical microscope image of the MTSM structure; (**c**) schematic illustration of the PDMS-coated MTSM structure.

Figure 1c illustrates the proposed PDMS-coated multimode-tapered single-mode-multimode (MTSM) structure. The fabrication flow is as follows: first, a multimode-single mode-multimode (MSM) fiber structure was fabricated by our self-developed precision cutting and splicing technique [27]. The two MMFs with a length of 300 µm were embedded into an SMF at a spacing of 1 mm. Then, the single-mode fiber was tapered by a fusion tapering technique, as shown in Figure 1a, and the fabricated tapered single-mode fiber was welded in two multimode fibers. The MSM structure was placed in the fusion tapering system (GPX-3000, Vytran, Morganville, NJ, USA) and both ends were fixed on the fiber holder. The spacing between the two MMFs was designed to be the same as the width of the heating unit (1 mm). The CCD device makes the middle SMF of the MSM coincide with the heating unit, thus ensuring that the heating unit can accurately heat the central region of the middle SMF. The direction, speed, and time of movement of the two displacement platforms during the heating process are controlled by a computer to change the diameter of the tapered fiber waist. Figure 1b presents an optical microscope image of the prepared MTSM structure. It can be observed from the figure that the lengths of the two MMFs are 300 µm, while the tapering length and waist diameter of the tapered SMF are 1.68 mm and 47 µm, respectively. Next, the PDMS precursor (Sylgard 184A, Dow Corning Corporation, Midland, MI, USA) and the curing agent (Sylgard 184B, Dow Corning Corporation, Midland, MI, USA) were mixed in a ratio of 10:1 and left to stand for 3 h. When the bubbles disappeared, we poured the prepared mixture into the mold so that the MTSM structure fixed in the mold was completely submerged. Finally, the mold containing the PDMS mixture and MTSM structure was heated to 50 °C for 2 h, and when the PDMS was fully cured, the PDMS-coated MTSM structure temperature sensor was successfully obtained.

For an MTSM structure, the two MMFs act as an optical splitter and an optical coupler, respectively. Because the diameter of the tapered fiber waist is greatly reduced, partial light will travel into the cladding to excite cladding modes, which will be re-coupled with the core mode in the second SMF–MMF welding zone, and finally generate the interference patterns [28,29]. When the phase difference is satisfied, the cladding modes will interfere with the core mode; the phase difference (*ϕ*) between the core mode and the high-order cladding modes after L distance propagation is written as:

(1) $$\phi =\frac{2\pi \Delta n_{eff}L}{\lambda }$$

where $\Delta n_{eff}=n_{eff}^{core}-n_{eff,m}^{clad}$ represents the effective refractive index difference of the core mode and the *m*-order cladding mode, *λ* is the wavelength in vacuum, and *L* is the length of the MTSM structure [30]. When *ϕ* = (2*k* + 1)π, the interference dip wavelength (*λ_k_*) will appear at:

(2) $$\lambda_{k}=\frac{2\left(n_{eff}^{core}-n_{eff,m}^{clad}\right)L}{2k+1}\;\;\;k=1,2,3......$$

Based on the above equations, the wavelength spacing (Δ*λ*) can be calculated by [31]:

(3) $$\Delta \lambda =\frac{\lambda^{2}}{\Delta n_{eff}L}$$

From Equation (3), it is clear that Δ*λ* is negatively correlated with Δ*n_eff_* and *L*. For the same Δ*λ*, the larger the difference in effective refractive index, the smaller the size of the MTSM structure. The interference intensity (*I*) is given by:

(4) $$I=I_{core}+I_{cladding}+2\sqrt{I_{core}I_{cladding}}cos\phi$$

where *I_core_* and *I_cladding_* represent the intensities of the core mode and the cladding mode, respectively. In order to obtain high-visibility interference fringes, the intensity difference between the core mode and the cladding mode needs to be smaller.

**Figure 2:**
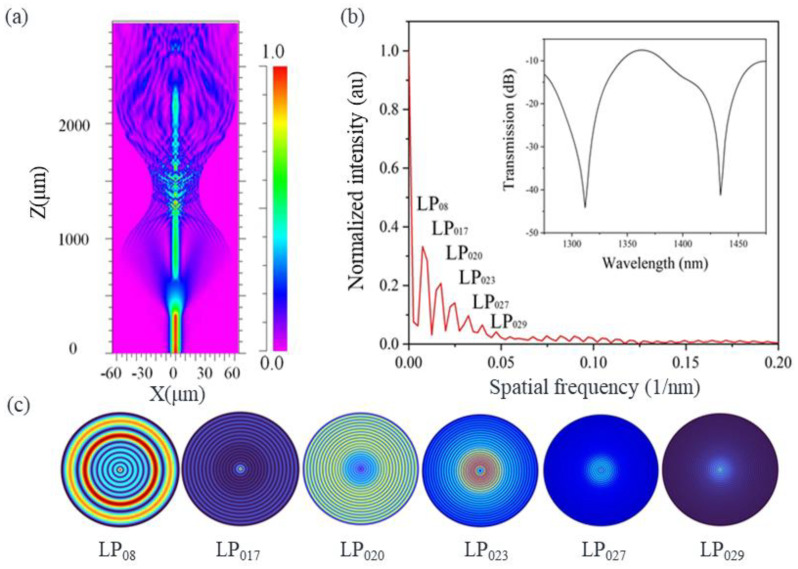
(**a**) Simulated optical field distribution of the bare MTSM structure; (**b**) spatial frequency spectrum of the bare MTSM structure. Inset is the corresponding transmission spectrum; (**c**) calculated cladding mode profiles.

We established a simulation model through the finite difference beam propagating method (FD–BPM) to analyze the cladding modes and the spectral properties [31]. The core/cladding refractive indexes (RIs) of the SMF and the MMFs are both 1.455/1.449 in the model. The total length of the MTSM structure is 2.28 mm and the core/cladding diameters of the MMFs are 60/125 µm. The simulated optical field distribution of the bare MTSM structure is shown in Figure 2a. It can be observed that the loss of the light field is mainly generated in the first SMF–MMF welding zone. Due to the large diameter difference between the tapered SMF and the MMF, when the light enters the tapered SMF from the first MMF, some of the light will travel forward in the core, while the rest of the light will travel into the cladding to produce cladding modes, which will be coupled with the core mode in the second SMF–MMF fusion zone, and finally generate the interference patterns [32]. In addition, we also use the fast Fourier transform (FFT) method to transfer the data of the transmission spectrum to the spatial frequency spectrum [33]. As shown in Figure 2b, the spatial frequency spectrum with high fringe visibility is extracted by the FFT method, and there are six clusters of frequency components in the FFT outputs. The inset in Figure 2b is a typical calculated spectrum obtained by the scanning wavelength method. The scanning wavelength interval and mesh size in the calculation are 0.2 nm and 0.02 µm, respectively. Figure 2c shows the calculated electric field distribution of the six cladding modes. From these mode field profiles, we can see that as the mode order increases, the electric field intensity gradually decreases. In these six cladding modes, LP_08_ and LP_017_ play a major role in constructing the interference spectrum.

**Figure 3:**
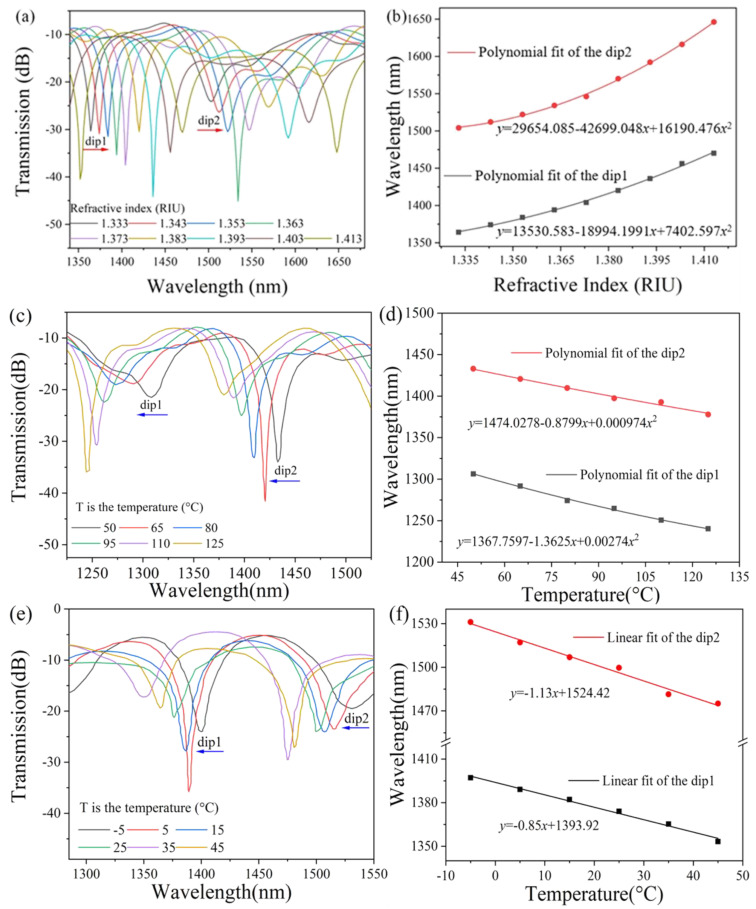
(**a**) Simulated transmission spectrum of the bare MTSM as the external RI increase; (**b**) resonant dips wavelength shifts as a function of RI; (**c**) simulated transmission spectra of the PDMS-coated MTSM as the external temperature increase (the temperature ranges from 50 °C to 125 °C); (**d**) resonant dips wavelength shifts as a function of temperature (the temperature ranges from 50 °C to 125 °C); (**e**) simulated transmission spectra of the PDMS-coated MTSM as the external temperature increase (the temperature range is from −5 °C to 45 °C); (**f**) resonant dips wavelength shifts as a function of temperature (the temperature ranges from −5 °C to 45 °C).

Figure 3a shows the simulated spectrum of the bare MTSM structure without PDMS coating corresponding to different external RIs. When the RI increases from 1.333 to 1.413, the interference fringes shift significantly towards a longer wavelength. The relationship between the resonance central wavelength and the external RI is shown in Figure 3b. As the external RI increases, the shift speed of the resonant dips wavelength also increases, and the RI sensitivity of the two resonant dips is 1925.54 nm/RIU (dip1) and 3055.24 nm/RIU (dip2) at RIU = 1.413. Because the TOC of quartz silica is low, the bare MTSM structure without PDMS coating is almost insensitive to changes in temperature. In view of this, we can obtain a high-sensitivity temperature sensor by coating the MTSM structure with a high TOC material (−4.66 × 10^−4^/°C) like PDMS [34]. The change in the RI of PDMS regarding temperature can be expressed as: RIU = 1.4176 − T × 4.5 × 10^−4^ [35,36]. From this formula, it is easy to calculate that as the temperature increases from 50 °C to 125 °C, the RI decreases correspondingly from 1.3951 to 1.361. The simulated spectra of the PDMS-coated MTSM structure corresponding to different external temperatures are shown in Figure 3c. When the temperature increases from 50 °C to 125 °C, the interference fringes shift significantly towards a shorter wavelength. The relationship between the resonance central wavelength and temperature is shown in Figure 3d. The temperature sensitivity of the two resonant dips is determined to be −0.65 nm/°C (dip1) and −0.87 nm/°C (dip2) at T = 50 °C. When the temperature increases from −5 °C to 45 °C, the interference fringes shift towards a shorter wavelength, as shown in Figure 3e. As illustrated in Figure 3f, the temperature sensitivity of the two resonant dips is determined to be −0.85 nm/°C (dip1) and −1.13 nm/°C (dip2). Due to longer wavelengths usually having a stronger evanescent field, the sensitivity of dip2 is higher than that of dip1.

## 3. Experimental Results and Discussion

**Figure 4:**
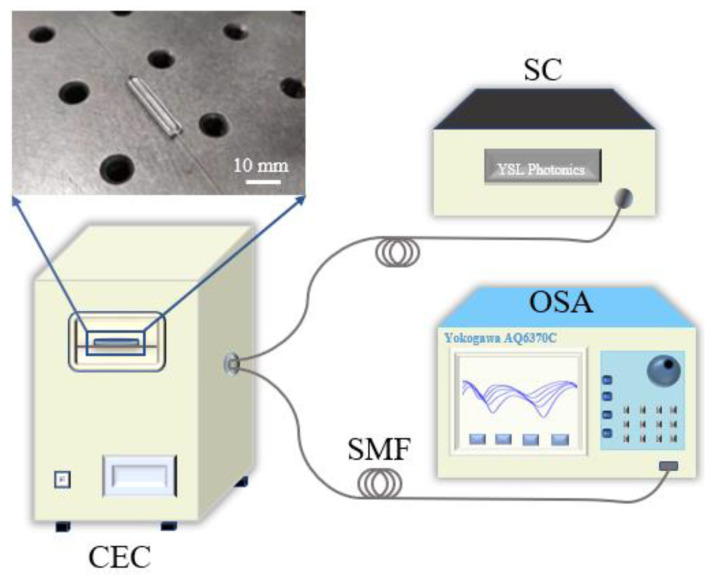
Schematic diagram of the temperature sensing setup.

The temperature sensing experimental setup is illustrated in Figure 4, which includes a super-continuous light source (SC), a controlled environmental chamber (CEC), and an optical spectrometer analyzer (OSA). The sensor was fixed on a glass slide and placed in a CEC. The two ends of the sensor were respectively connected with the SC (YSL Photonics, Wuhan, China, SC-5) and the OSA (Yokogawa, Tokyo, Japan, AQ6370C) with a wavelength resolution of 0.01 nm. In this temperature sensing test, the CEC was used to regulate the environmental temperature range from −5 °C to 125 °C with a temperature resolution of 0.1 °C. In order to make the measurement more accurate, it is necessary to maintain the temperature for 10 min before the next test. The corresponding transmitted spectra are recorded in the optical spectrometer, and the final sensitivity of the temperature sensors can be obtained by analyzing spectral data at different temperatures.

**Figure 5:**
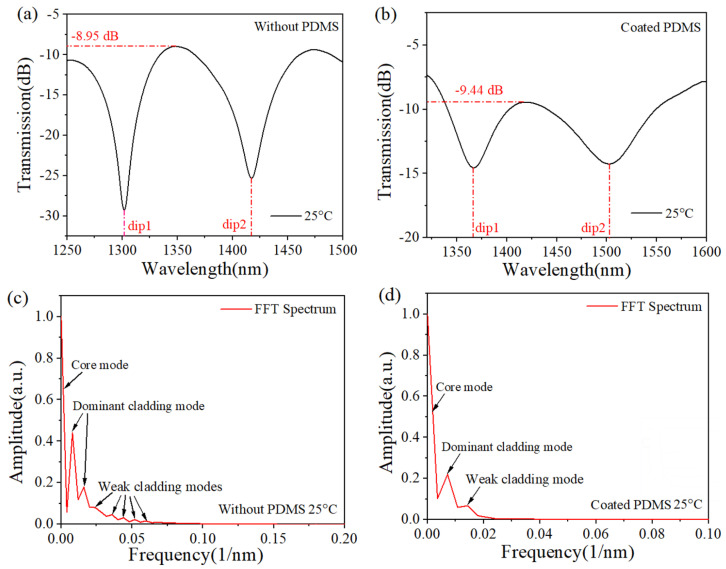
Transmission spectrum (**a**) without PDMS and (**b**) with PDMS at 25 °C; fast Fourier transform (FFT) spectrum (**c**) without PDMS and (**d**) with PDMS at 25 °C.

Figure 5 shows a comparison of the spectra before and after coating the sensor with PDMS, as well as a comparison of the FFT spectra. Figure 5a,b show the transmission spectra of the device when the MTSM was surrounded by air and PDMS at room temperature (25 °C), respectively. The effective refractive index difference (Δ*n_eff_*) of the core mode and the *m*-order cladding mode increases due to the mode-coupling effect. According to Equation (1), the phase difference (*ϕ*) is constant, and the wavelength shifts towards longer wavelengths. The insertion loss of the MTSM is estimated to be −8.95 dB. Upon coating the MTSM surface with PDMS, the insertion loss rises marginally to −9.44 dB. Figure 5c shows that the spatial spectrum comprises core modes, prominent excitation cladding modes, and several less intense excitation cladding modes. As a result, the main interference occurs between the two dominant excitation cladding modes and the core modes. The contribution of the weaker cladding modes to the interference pattern is negligible because of their relatively low intensity. In Figure 5d, we found that one main cladding mode and weak cladding mode were involved in the interference. Comparing Figure 5c,d, when multiple modes interfere in the tapered region, the light intensity loss of the cladding mode is small, resulting in a larger fringe intensity. After the coating of PDMS, the PDMS with refractive index *n* = 1.42 becomes the cladding layer in the tapered region instead of the air (*n* = 1), and part of the energy of the cladding mode can escape to the PDMS layer, resulting in less energy participating in the interference, thus inhibiting the excitation of the cladding mode.

**Figure 6:**
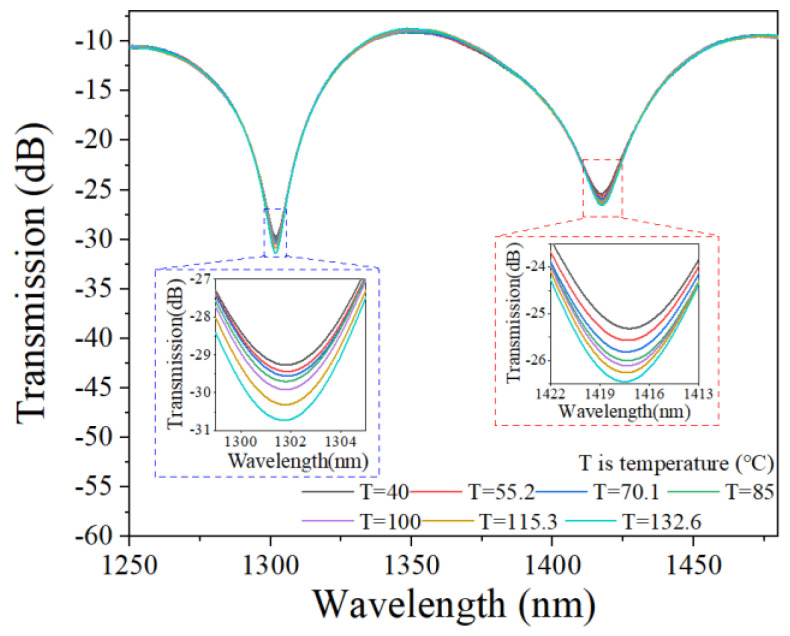
Transmission spectra of the bare MTSM structure at different temperatures.

The temperature-sensing characteristics of the bare MTSM were then investigated. The transmitted spectra of the bare MTSM structure without PDMS coating at different temperatures are shown in Figure 6. When the external temperature increases, the resonant dips have almost no change and the contrast depth is less than 2 dB. It can be seen from the spectral curves that the bare MTSM structure without PDMS coating is almost insensitive to changes in temperature due to the low TOC of quartz silica.

**Figure 7:**
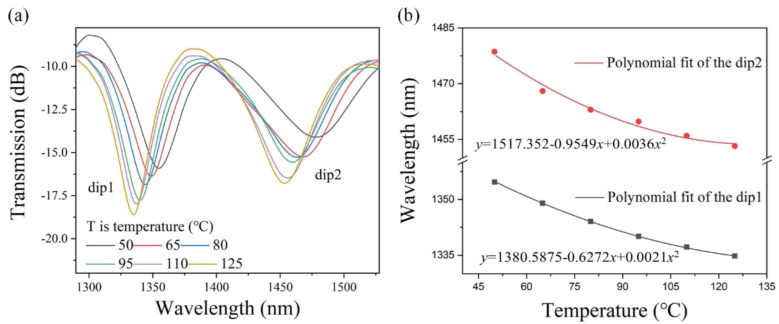
(**a**) Transmission spectral evolution of the PDMS-coated MTSM in the range of 50~125 °C; (**b**) resonant dips wavelength shifts versus temperatures.

Then, the temperature-sensing properties of the PDMS-coated MTSM were examined. The experimental spectra of the PDMS-coated MTSM structure during the external temperature increase from 50 °C to 125 °C are shown in Figure 7a. When the external temperature increases from 50 °C to 125 °C, the central wavelength of the resonant dips shifts significantly to the short wavelength (blue shift). This phenomenon is mainly due to the high TOC of PDMS, which makes the PDMS-coated MTSM structure sensitive to temperature changes. The change in temperature causes the change of the effective refractive index to form the interference depression wavelengths and, finally, causes the blue shift of the resonance central wavelength. The shifts in central wavelength caused by temperature variations are plotted and polynomially fitted, as shown in Figure 7b. It can be observed that the shift velocity of resonant dips wavelength decreases with the increase of the external temperature, and the sensitivity of dip1 and dip2 can reach −0.42 nm/°C and −0.59 nm/°C, respectively.

**Figure 8:**
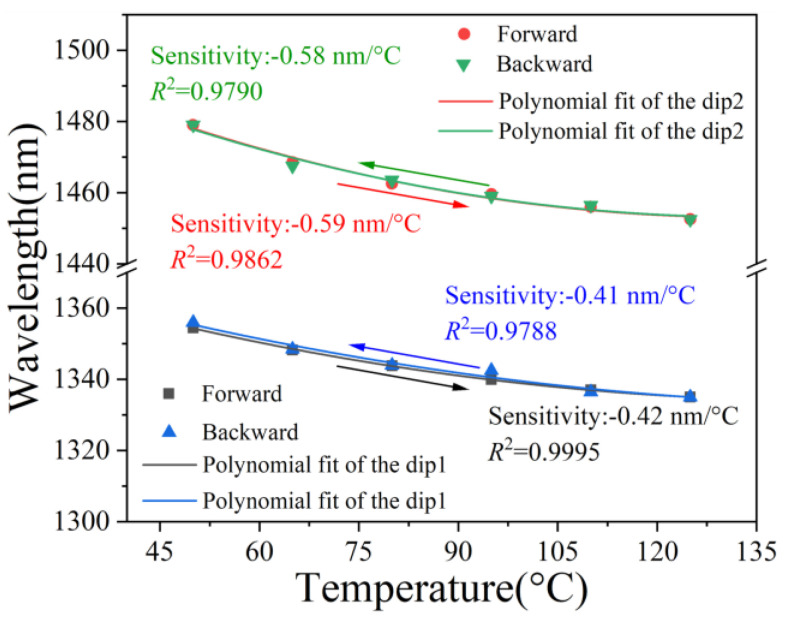
Temperature repeatability performances of the sensor.

In the repeatability experiment, the temperature in the CEC gradually increased from 50 °C to 125 °C and then progressively reduced to 50 °C. The temperature repeatability of the sensor is shown in Figure 8. It is observed that the intersection of internal envelopes exhibits a polynomial shift in response to temperature variations, and the temperature sensitivities were approximately the same during the temperature increase and decrease (*R*^2^ > 0.9788).

**Figure 9:**
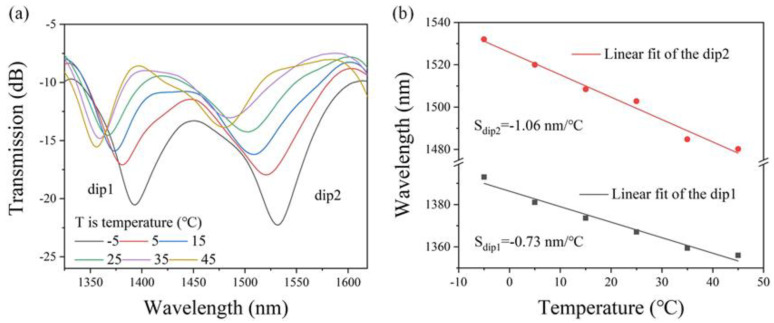
(**a**) Transmission spectral evolution of the PDMS-coated MTSM in the range of −5~45 °C; (**b**) resonant dips wavelength shifts versus temperatures.

Figure 9a shows the transmission spectral evolution of the PDMS-coated MTSM structure during the external temperature increase from −5 °C to 45 °C. It is consistent with the temperature sensing results in the high-temperature range (50 °C to 125 °C). The resonance wavelength shifts significantly to the short wavelength (blue shift) with the increase of the external temperature. From the spectra, we can also see that the central wavelength of resonant dips shifts regularly and is easy to distinguish and analyze and that the sensitivity of the proposed sensor performs better in the low-temperature range than in the high-temperature range. The shifts in central wavelength caused by temperature variations were plotted and linearly fitted, as shown in Figure 9b. When the external temperature increases from −5 °C to 45 °C, the central wavelength of the resonant dips shifts towards the shorter wavelength with a linear coefficient of 98.93%. This proposed temperature sensor has a maximum sensitivity of −0.73 nm/°C and −1.06 nm/°C for dip1 and dip2, respectively. Because the cladding mode of the longer wavelength is more susceptible to variations of the surrounding RI, dip2 shows better sensitivity than dip1. Furthermore, we also carried out this temperature sensing experiment several times to assess the stability of the sensor, and the results of these experiments confirm that the temperature sensor is reliable in terms of stability.

**Figure 10:**
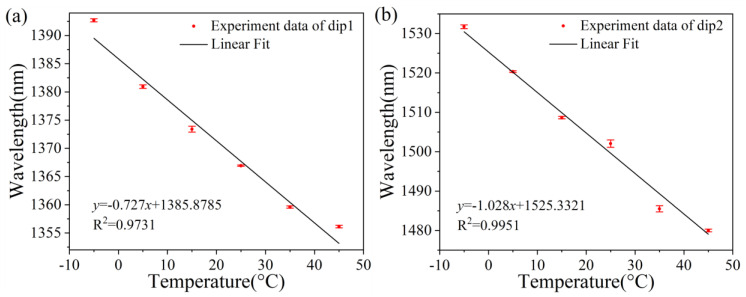
Repetitive experimental results of measuring temperature using (**a**) Dip 1 and (**b**) Dip 2, respectively.

In order to show the good repeatability of the sensor more intuitively, the repeated data in Figure 10a,b were linearly fitted. According to the repeated experimental results of temperature sensing, it can be seen that in three repeated experiments, the error bars at maximum dip1 and dip2 are 0.529 nm and 0.945 nm, respectively. The errors of data collection points are relatively small. Therefore, it can be concluded that the prepared sensor has excellent sensitivity and good performance repeatability.

**Figure 11:**
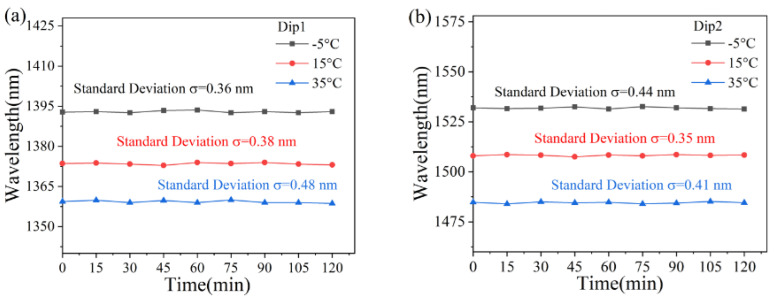
Stability experimental results of measuring temperature using (**a**) Dip 1 and (**b**) Dip 2, respectively.

The wavelength changes of the three dips were recorded nine times at −5 °C, 15 °C, and 35 °C every 15 min. As illustrated in Figure 11, the experimental findings emphasize the sensor’s remarkable temperature stability. Figure 11a,b effectively showcase the stable behavior of dip1 and dip2. During the 0 to 120-min interval, the interferometric spectra show subtle variations that can be attributed to minor equipment deviations. Notably, the standard deviations at each temperature measurement stay reliably below 0.48 nm, falling well within the permissible fluctuation limits. This level of temperature stability is a clear indicator of the sensor’s superior performance and dependability.

**Figure 12:**
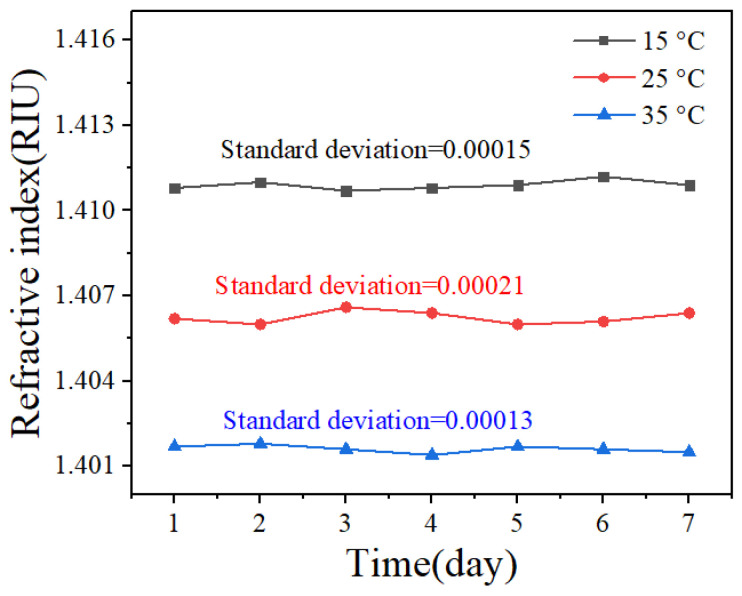
Stability experimental results of measuring the polydimethylsiloxane refractive index.

The stability of the polydimethylsiloxane refractive index was evaluated at temperatures of 15 °C, 25 °C, and 35 °C. The testing period lasted 7 days, during which data were recorded at 4-h intervals and daily averages were subsequently calculated. The maximum standard deviation is 0.00021 RIU, as shown in Figure 12.

In Table 1, we compare the performances of fiber temperature sensors with different fiber configurations. The previous uncoated MZI sensors show relatively low temperature sensitivity [37,38,39]. The temperature sensors’ dependence on temperature-sensitive materials [21,22,26,40,41,42,43,44] have high sensitivities; however, in these temperature sensors, overlong sensing units limit their applications. Compared to these previous reports, the proposed temperature sensor based on PDMS-coated MTSM has a relatively high temperature sensitivity and a smaller size.

## 4. Conclusions

In this paper, we have demonstrated a highly sensitive temperature sensor based on an MZI coated by PDMS film. The MZI was fabricated by embedding a tapered single-mode fiber between two multimode fibers. When the temperature changes, the thermo-optical effect of PDMS causes the central wavelength of the resonant dips to shift. The transmission spectra and temperature sensitivities of the proposed sensor have been studied by theoretical simulation and experimental verification. Simulated and experimental results indicate that the temperature sensitivity of the sensor is as high as −1.06 nm/°C in the range from −5 °C to 45 °C. Due to their simple fabrication process and compact construction size, such sensors provide an inexpensive and practical building block for achieving highly sensitive temperature sensing.

## Figures and Tables

**Table 1:** Characteristics of temperature sensors based on various fiber structures.

Structure	Size (mm)	Range (°C)	Sensitivity (pm/°C)	Whether Temperature-Sensitive Materials Are Required	Ref.
Two-fiber taper	54.5	20–60	77	No	[37]
HCF and FMF	50.037	0–120	−38.8	No	[38]
D-shaped no-core fiber	40	25–80	86.1	No	[39]
SHF	80	23–90	105	Yes (alcohol)	[21]
EHADCF	10.5	10–100	−562.4	Yes (matching liquid)	[22]
Tapered HCF	39	32–46	−3232.1	Yes (PDMS)	[40]
Mismatch structure of three SMFs	40	20–100	101	Yes (PDMS)	[41]
TCF–NCF–TCF	30	45–80	130	Yes (PDMS)	[42]
PDMS-coated MZI combined with an FBG	2.34	30–60	11,190	Yes (PDMS)	[43]
PCFI	20	35–65	−255	Yes (PDMS)	[26]
TCF	8	40–70	166.8	Yes (PDMS)	[44]
STMS	2.28	−5–45	−1060	Yes (PDMS)	This work

## Data Availability

The data will be available upon request.
